# Newcastle disease virus-induced autophagy mediates antiapoptotic signaling responses *in vitro* and *in vivo*

**DOI:** 10.18632/oncotarget.18169

**Published:** 2017-05-25

**Authors:** Yinfeng Kang, Runyu Yuan, Bin Xiang, Xiaqiong Zhao, Pei Gao, Xu Dai, Ming Liao, Tao Ren

**Affiliations:** ^1^ College of Veterinary Medicine, Key Laboratory of Zoonosis Prevention and Control of Guangdong Province, South China Agricultural University, Guangzhou, 510642, China; ^2^ State Key Laboratory of Oncology in South China, Collaborative Innovation Center for Cancer Medicine, Department of Experimental Research, Sun Yat-sen University Cancer Center, Guangzhou, 510060, China; ^3^ Key Laboratory of Animal Vaccine Development, Ministry of Agriculture, Guangzhou, 510642, China; ^4^ Key Laboratory for Repository and Application of Pathogenic Microbiology, Research Center for Pathogens Detection Technology of Emerging Infectious Diseases, Guangdong Provincial Center for Disease Control and Prevention, Guangzhou, 511430, China

**Keywords:** Newcastle disease virus, autophagy, apoptosis, relationship, replication

## Abstract

In this study, we investigated the role of autophagy and apoptosis in Newcastle disease virus (NDV)-infected chicken cells and tissues. NDV-infected and starvation-induced chick embryo fibroblasts (CEF) cells showed higher autophagosome formation than mock-infected CEF cells on transmission electron microscopy. The NDV-infected CEF cells showed enhanced conversion of microtubule-associated protein 1 light chain 3-I (LC3-I) to LC3-II and degradation of p62/SQSTM1. The diminished conversion of LC3-I to LC3-II and cleaved caspase 3 and poly (ADP-ribose) polymerase (PARP) in ultraviolet-inactivated NDV-infected cells suggested that autophagosome formation was necessary for NDV replication. Inhibition of autophagy by chloroquine (CQ) enhanced apoptosis resulting in increased cleavage of caspase 3 and PARP and AnnexinV/propidium iodide staining. Autophagy induction by rapamycin resulted in upregulation of all autophagy-related genes except Beclin 1, anti-apoptosis factors, and proinflammatory cytokines in the NDV-infected spleen and lung tissues. Subsequently, decreased apoptosis was observed in NDV-infected spleens and lungs than mock-infected organs. The pan-caspase inhibitor ZVAD-FMK promoted conversion of LC3-I to LC3-II, the degradation of p62/SQSTM1, NDV replication and cell viability by inhibiting apoptosis. Our study demonstrates that apoptosis inhibition enhances autophagy and promoted cell survival and NDV replication.

## INTRODUCTION

Newcastle disease virus (NDV), the causative agent of Newcastle disease (ND), is an enveloped, single-stranded, non-segmented, negative-sense RNA virus of the Paramyxoviridae family that causes enormous economic losses to various avian industries worldwide [1]. The NDV genome is approximately 15kb long that encodes six structural proteins, fusion protein (F), hemagglutinin–neuraminidase protein (HN), nucleocapsid protein (NP), matrix protein (M), phosphoprotein (P), and large polymerase protein (L) and two non-structural proteins, V and W, which are produced by RNA editing during P gene transcription as part of the viral life cycle [2]. Although compulsive culling and vaccination strategies have greatly reduced the NDV outbreaks, it continues to pose significant health problems to world’s avian industries [3].

Cell death in multicellular organisms is classified into apoptosis, autophagy, and necrosis, each of which are morphologically distinct [4]. Apoptosis is a physiologically programmed cell death that is essential for eliminating infected or damaged cells in order to maintain cellular homeostasis and limit disease pathogenesis [5, 6]. The cell morphological features and biochemical characteristics of apoptosis include DNA fragmentation, cytoplasmic vacuoles, membrane blebbing and formation of apoptotic bodies [6, 7]. Apoptosis can be triggered either intrinsically via mitochondrial outer membrane permeabilization or extrinsically via death receptor oligomerization [8, 9]. In both cases, the cysteine aspartyl-specific proteases or caspases (e.g., caspase-3, −8, and −9) are activated that trigger morphological and biochemical changes that are characteristic of apoptosis. However, the mechanisms of caspase activation remain distinct. Apoptosis is the first line of cellular defense against pathogen infection that severely attenuates viral proliferation by activation of intracellular stress pathways, thereby resulting in cell death [10, 11]. Several viruses like the influenza virus, simian virus 40, herpes simplex virus type 1, human papillomaviruses, and human adenoviruses strategically delay or evade early-stage apoptosis, thereby prolonging the life of the infected cells, thereby helping viral replication [12].

In contrast, autophagy is a homeostatic biological process in which cytoplasmic components like macromolecules (e.g., viruses, proteins, and bacteria) and organelles (e.g., endoplasmic reticulum, golgi apparatus, and mitochondria), are degraded by proteasomes or recycled in lysosomes [13]. The autophagy processes degrade damaged cell components, invading microorganisms and disused or defunct organelles, and also mediate inflammation and immune responses [13, 14]. Although autophagy is triggered by cellular stress (e.g., starvation, hypoxia, and pathogenic stimuli), the mechanisms of autophagy-mediated innate and acquired immunity are not well characterized. Autophagy plays a crucial role in host defense responses and is critical for cell survival, T cell priming, the production of type I interferons, and major histocompatibility complex (MHC) I or II antigen presentation to CD4+ cells. Many viruses exploit the autophagic processes for replication, survival, and dissemination in host infected cells. For example, the influenza A virus M2 and the dengue virus NS1 proteins inhibit the fusion of autophagosomes and lysosomes [15, 16]; the human immunodeficiency virus encodes the Nef protein that inhibits the acidification of autophagosomes [17]; and the hepatitis C virus, which expresses the NS4B protein, forms a complex with Rab5 and Vps34 to inhibit the formation of autophagosomes [18].

Although previous studies have demonstrated that NDV infection induces apoptosis and autophagy [19–23], their role during NDV infection is unknown. Therefore, the objective of our study was to investigate the roles of apoptosis and autophagy during NDV infection.

## RESULTS

### Newcastle disease virus induces autophagosomes in chicken cells

**Figure 1:**
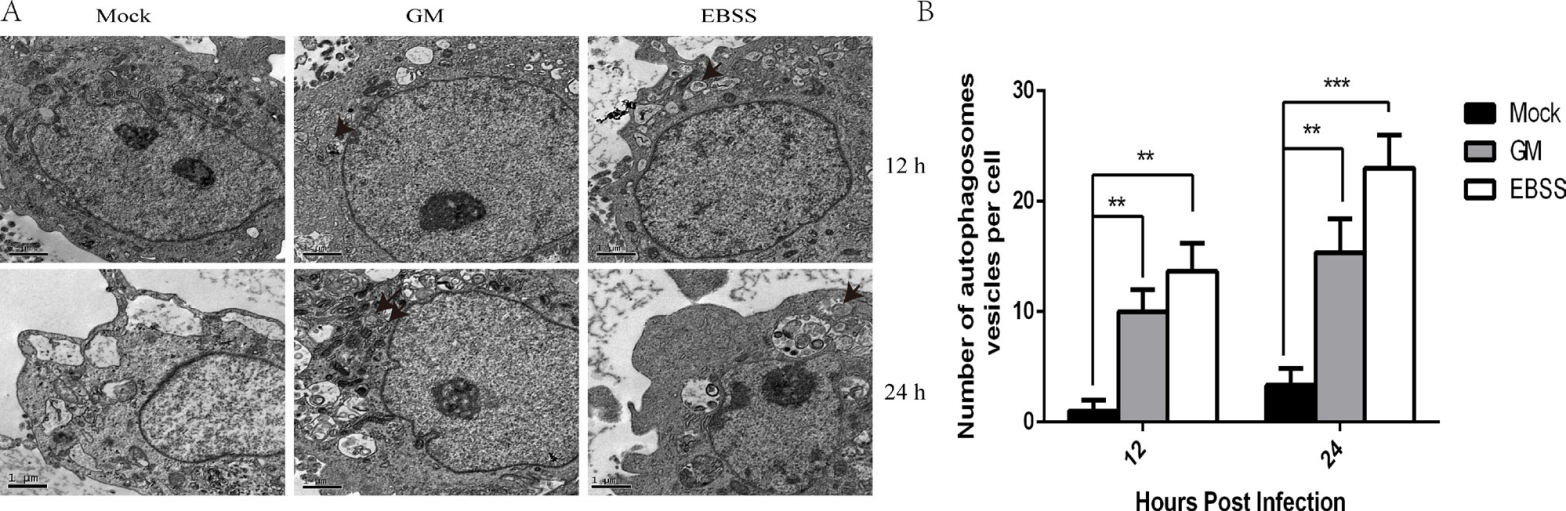
Autophagosome formation in Newcastle disease virus (NDV) chick embryo fibroblast (CEF) cells (**A**) CEF cells were infected with NDV at a multiplicity of infection of 1 or treated with EBSS (i.e., starvation) for 1h and subjected to transmission electron microscopy at 12 and 24 h post-infection to observe autophagosomes (indicated by black arrows). (**B**) Autophagosomes per cell from (A) were calculated; data represent *Mean* ± *SD* for triplicate samples of at least 100 cells per sample. Similar results were obtained from three independent experiments; **p < .01, ***p < .001 (*t* test).

The autophagic vacuoles (autophagosomes) is a hallmark feature of autophagy [14, 24]. First, we infected CEF and DF-1 cells with NDV at an MOI of 1 and analyzed autophagosome formation at 12 and 24 h using TEM. CEF and DF-1 cultures without viral incubation were used as negative controls, whereas EBSS treated CEF and DF-1 cultures were used as positive controls. As shown in Figure 1, we observed increased single- and double-membrane bound structures in NDV and EBSS treated CEF cells at 12 and 24 h compared to negative controls. Similar results were observed in DF-1 cells (data not shown).

**Figure 2:**
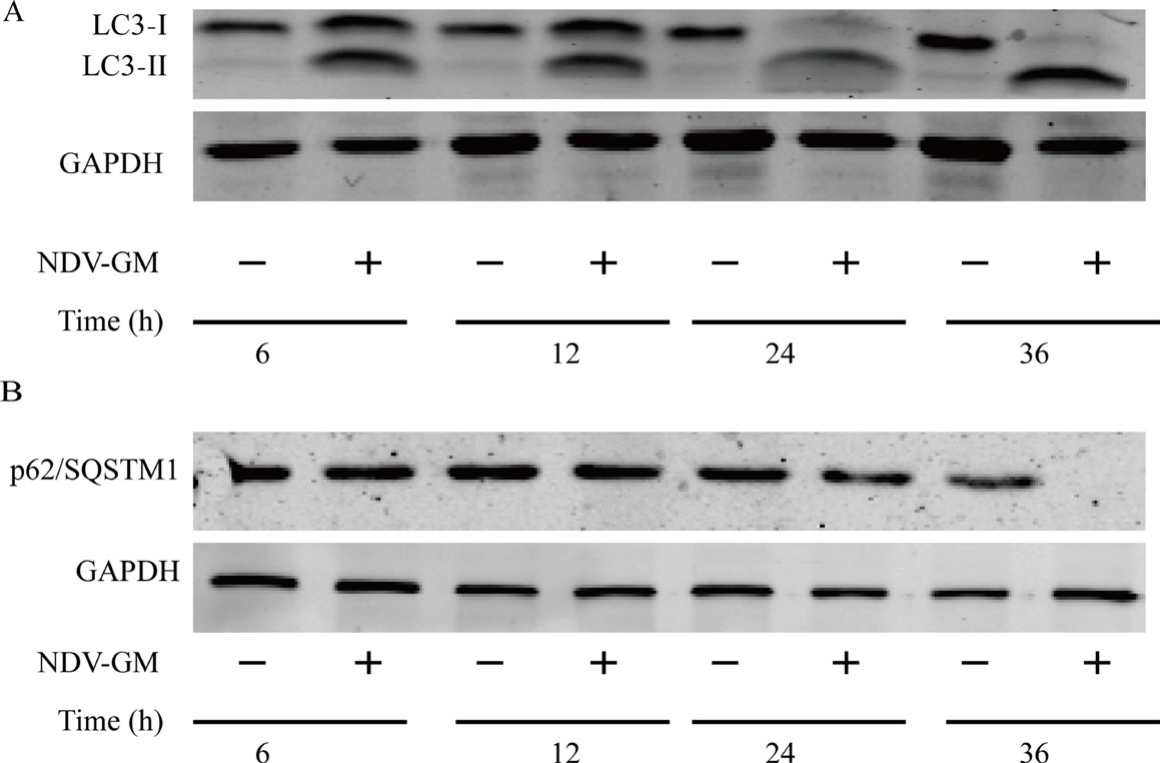
Newcastle disease virus (NDV) infection induces the formation of LC3 puncta (**A**) and preserves autophagic flux (**B**) in chick embryo fibroblast (CEF) cells. CEF cells were infected with NDV at a multiplicity of infection of 1 and western blot analysis was performed with anti-LC3B and anti-p62/SQSTM1 antibodies at 6, 12, 24 and 36 h post-infection. Anti-GAPDH antibody was used as an endogenous control for protein loading.

Autophagy is mediated by ATG proteins such as microtubule-associated protein 1 light chain 3 (LC3) that play distinct roles in different stages of autophagosome formation [25]. Therefore, we analyzed LC3 and p62/SQSTM1 to verify the status of autophagy. CEF cells were infected with NDV at an MOI of 1 and analyzed at 6, 12, 24, and 36h by western blot to detect conversion of LC3 and the degradation of p62/SQSTM1. As shown in Figure 2A, the conversion of LC3-I to LC3-II induced by NDV was greater than in mock-infected cells, especially at the later stages of infection. Also, NDV infection increased the degradation of p62/SQSTM1 in CEF cells over time (Figure 2B). Similar results were obtained for NDV-infected DF-1 cells (data not presented). These data suggested that NDV strain GM induced autophagy in chicken cells.

### Inhibition of autophagy results in apoptosis

**Figure 3:**
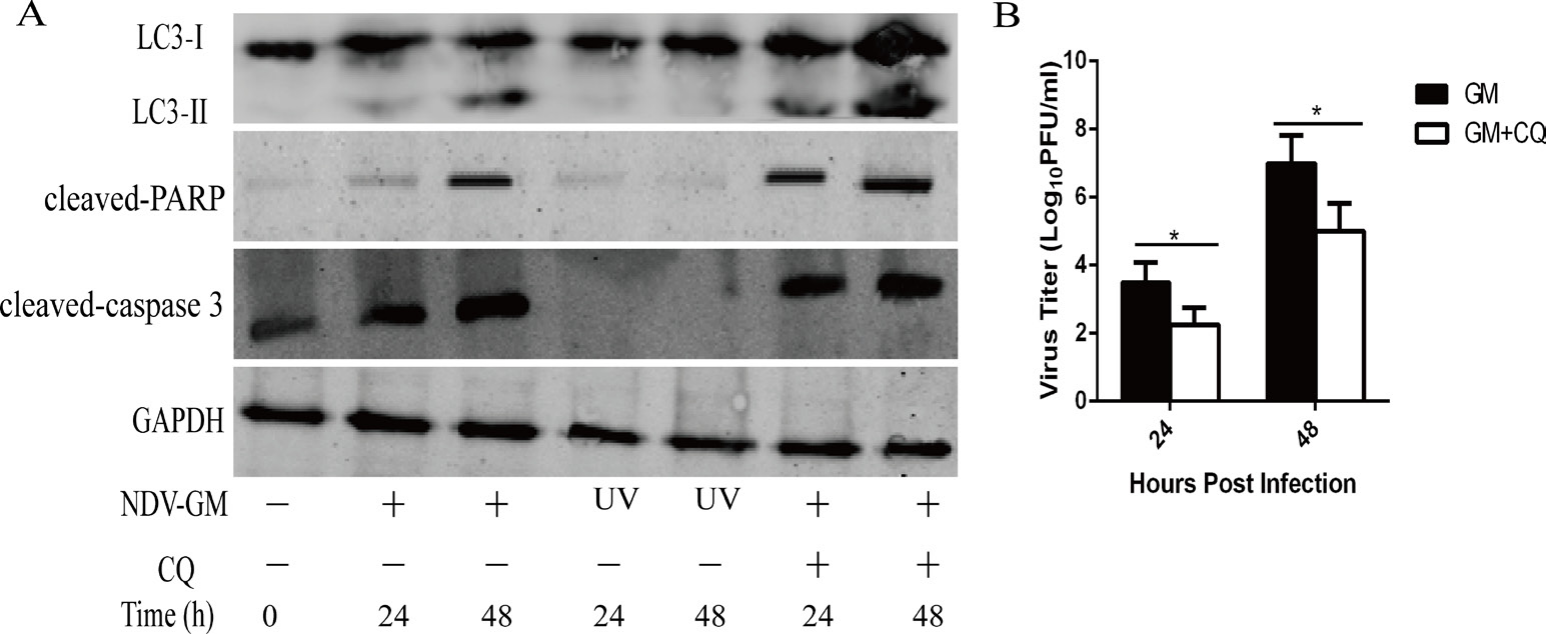
Effect of chloroquine (CQ) on autophagy, apoptosis and NDV replication in NDV-infected CEF cells (**A**) CEF cells were preincubated with 50 µM CQ for 1 h followed by infection with NDV at a multiplicity of infection of 1, after which cell lysates and culture supernatant were harvested for western blot analysis and (**B**) viral titration by plaque assay at 24 and 48 h postinfection, respectively. Mock-infected and UV-inactivated NDV-infected CEF cells were used as controls.

**Figure 4:**
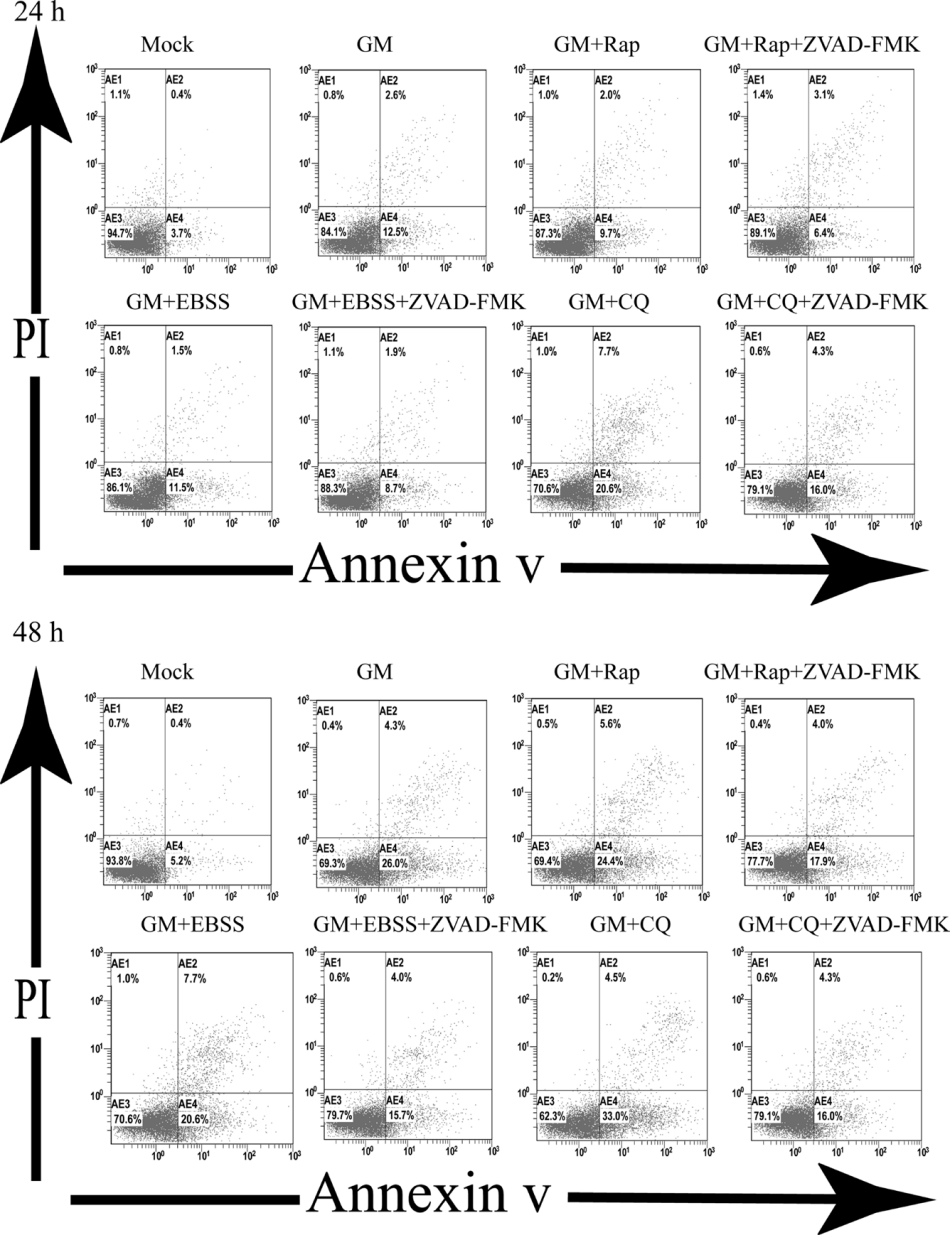
Autophagy regulates apoptosis in NDV-infected CEF cells CEF cells were pretreated with chloroquine (50 µM), rapamycin (100 µM), or Earle’s Balanced Salt Solution (starvation) in the presence or absence of pan-caspase inhibitor ZVAD-FMK (40 μM). Then, the cells were infected with NDV at a MOI of 1 and harvested and stained with annexinV and propidium iodide (PI) at indicated times. Stained cells were analyzed by flow cytometry. AnnexinV-positive and PI-negative cells in the lower right quadrant were counted as apoptotic cells.

Chloroquine (CQ) is an inhibitor of autophagy by reducing the acidity of lysosomes and inhibiting the fusion of autophagosomes and lysosomes [26]. To elucidate the role of autophagy in NDV-infected chicken cells, CEF cells were pretreated with 50 µM CQ for 1 h, infected with NDV (MOI = 1), and analyzed at indicated time points by western blot. The cell culture supernatants were also harvested to detect viral titers by plaque assay at 24 and 48h. As previous studies had shown [20], CQ increased the conversion of LC3-I to LC3-II in CEF cells, but decreased viral yield (Figure 3A and 3B). Moreover, the inhibition of autophagy increased the cleavage of key apoptotic proteins, caspase 3 and PARP at 24 and 48 h (Figure 3A). Furthermore, CEF cells infected with uv-inactivated NDV, which inhibits virus replication without affecting the interaction of NDV and its receptors (e.g., sialic acid receptor), inhibited conversion of LC3-I to LC3-II and the cleavage of caspase 3 or PARP at 24 and 48 h (Figure 3A). To further confirm that the inhibition of autophagy by CQ promoted NDV-activated apoptosis in CEF cells, we analyzed CEF cells that were pretreated with CQ (50 µM) prior to viral infection with NDV (MOI = 1) by annexin V–FITC/propidium iodide double-staining flow cytometry assay at 24 and 48 h. We observed that NDV-infected CEF cells with CQ (20.6 to 33%) showed increased apoptosis compared to NDV-infected CEF cells without CQ (12.5 to 26%) at 24 to 48 h (Figure 4). Therefore, our results demonstrated that autophagy inhibition enhanced apoptosis in NDV-infected chicken cells.

### Beclin 1 knockdown enhances apoptosis and decreases viral progeny yield

Beclin 1, the mammalian orthologue of yeast Atg6, plays a crucial role in autophagy and programmed cell death in mammalian systems [27, 28]. We investigated the role of autophagy in apoptosis and NDV replication by downregulating Beclin 1 by RNAi. RNAi of Beclin 1 resulted in decreased conversion of LC3-I to LC3-II, Beclin 1 and the viral titers, but increased the cleavage of PARP and caspase 3 at 24 and 48 h compared to scrambled control siRNAs. These data demonstrated that Beclin 1 was required for autophagy and NDV replication, whereas basal Beclin 1 levels were sufficient to initiate apoptosis.

### Induction of autophagy reduces apoptosis

**Figure 5:**
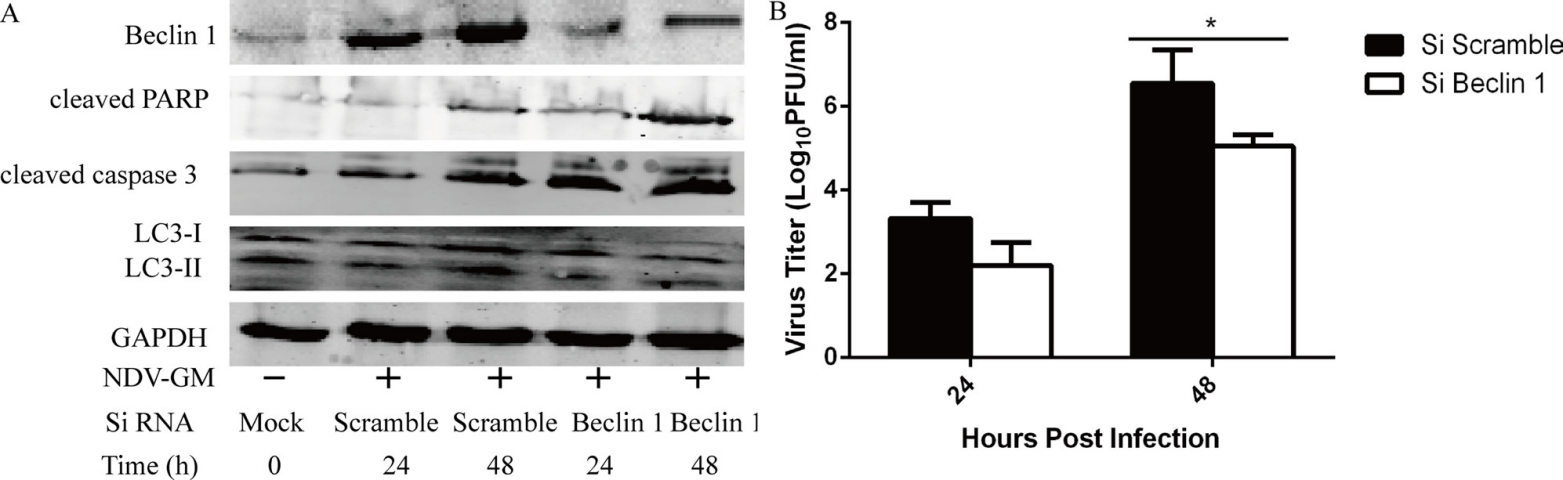
Inhibition of autophagy by Beclin1 knockdown enhances apoptosis but reduces NDV replication in CEF cells CEF cells were transfected with Beclin1-specific or control scrambled siRNAs for 24 h followed by NDV infection at a MOI of 1. Cell lysates and culture supernatant were harvested and subjected to western blot analysis (**A**) and viral titration by plaque assay (**B**) at 24 and 48 h post-infection, respectively. *Mean* ± *SD* of three independent experiments are shown; *p < .05 (*t* test).

**Figure 6:**
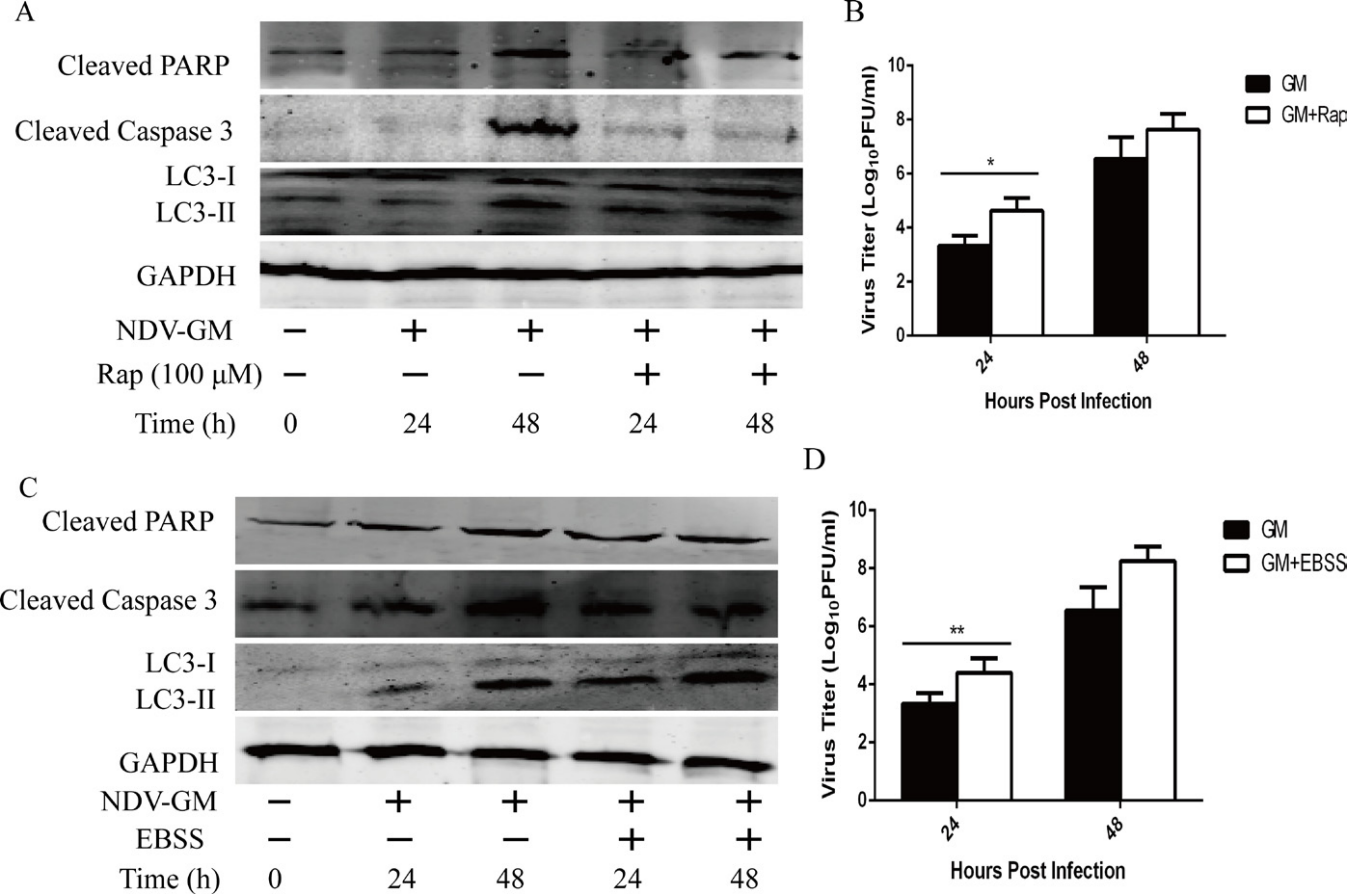
Induction of autophagy by rapamycin (Rap) or Earle’s balanced salt solution (EBSS) blocks apoptosis but promotes NDV replication in CEF cells CEF cells were pretreated with Rap (100 µM) or EBSS ( starvation) for 1h followed by infection with NDV at a MOI of 1. Cell lysates and culture supernatants were harvested and subjected to western blot analysis (**A** and **C**) and viral titration by plaque assay (**B** and **D**) at 24 and 48 h post-infection, respectively. *Mean* ± *SD* of three independent experiments are shown; *p < .05, **p < .01 (*t* test).

To further define the relationship between autophagy and apoptosis, CEF cells were pretreated with rapamycin (Rap), an inhibitor of mTORC1 that prevents the accumulation of autophagosomes and induces autophagy; and EBSS, which induces autophagy by nutrient starvation [14]. CEF cells were pretreated with 100 µM Rap or EBSS prior to viral infection with NDV at an MOI of 1. At 24 and 48 hpi, cells were harvested for western blot and flow cytometry analyses, and cell culture supernatants were collected to determine viral titers by plaque assay. As shown in Figure 6A and 6B, Rap treatment increased both the conversion of LC3-I to LC3-II and the NDV viral titers, but decreased the cleavage of PARP and caspase 3. Further, we analyzed apoptosis using annexinV–FITC double-staining flow cytometry assay at 24 and 48 h. We observed decreased apoptosis of NDV-infected CEF cells with Rap treatment compared to NDV-infected CEF cells without Rap (Figure 5). Similar results were obtained with EBSS treatment (Figure 6C and 6D). Overall, our results indicated that the induction of autophagy promoted virus replication but decreased apoptosis in host cells.

### Inhibition of apoptosis enhances autophagy and cell survival

**Figure 7:**
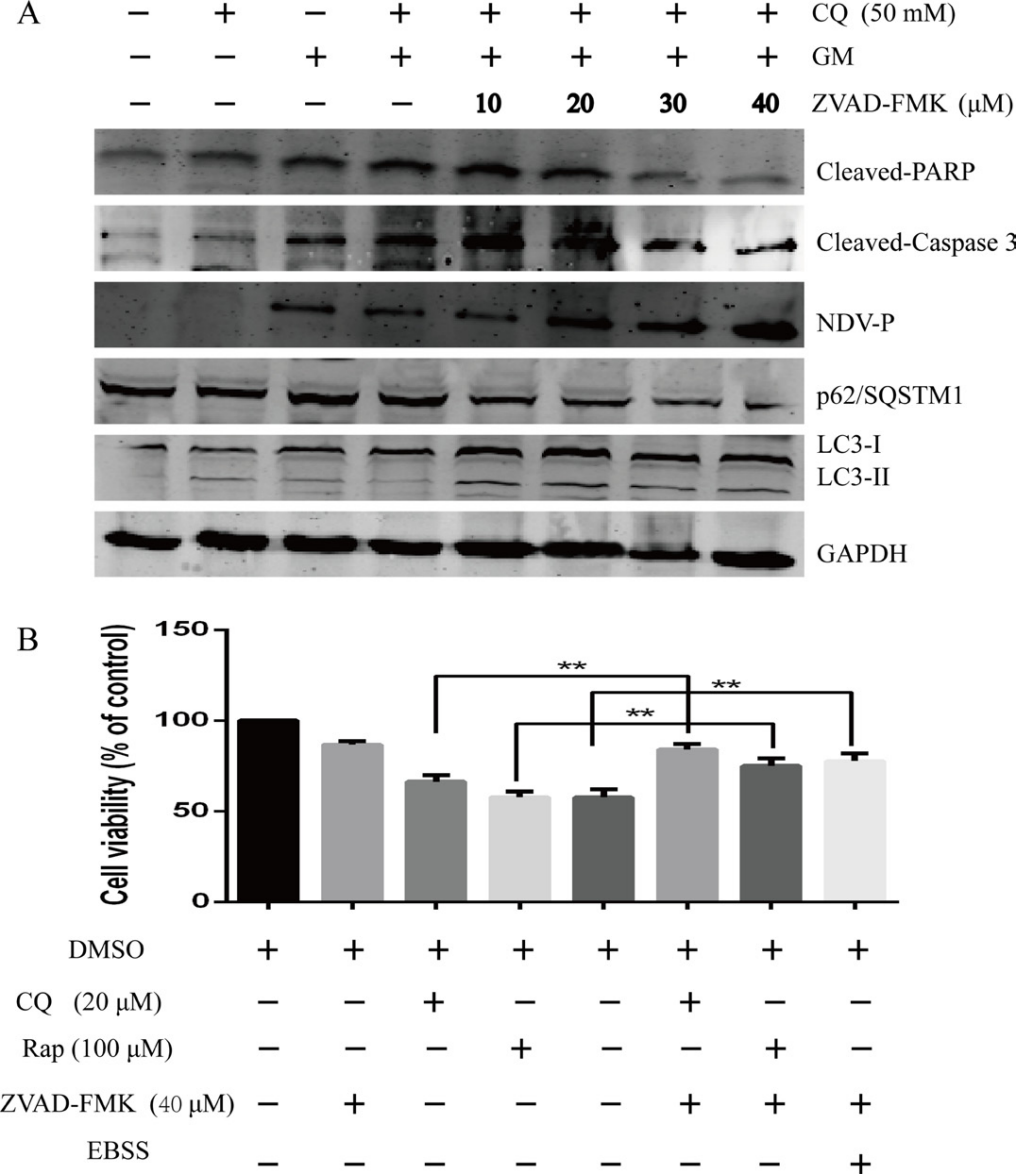
Inhibition of apoptosis precedes the enhanced induction of autophagy and the NDV-stimulated survival pathway (**A**) CEF cells were pretreated with increasing concentrations of ZVAD-FMK (10, 20, 30, and 40 µM) in the presence or absence of chloroquine (CQ, 50 µM), followed by NDV infection at a MOI of 1 for 1 h. At 24 h post-infection, cell lysates were subjected to western blot analysis. (**B**) CEF cells were incubated with Z-VAD-FMK (40 µM) in the presence of dimethyl sulfoxide with or without CQ (50 µM), rapamycin (100 µM), or Earle’s Balanced Salt Solution (starvation) for 24 h. Cell viability was determined by methylthiazolyldiphenyl-tetrazolium bromide (MTT) assay. *Mean* ± *SD* of three independent experiments are shown; **p < .01 (*t* test).

To investigate the role of apoptosis in NDV-induced autophagy and cell survival, we used a pan-caspase inhibitor, ZVAD-FMK that blocks caspase-dependent apoptosis [29]. We preincubated CEF cells with increasing concentrations of ZVAD-FMK (10, 20, 30, and 40 μM) in the presence or absence of CQ (50 μM) and then infected the cells with NDV (MOI = 1). After 24 h, we analyzed cell lysates by western blot. Treatment with CQ (50 μM) prior to NDV infection increased the cleavage of PARP and caspase 3 (Figure 7A). However, treatment with ZVAD-FMK prior to NDV infection increased the conversion of LC3-I to LC3-II, the level of the NDV-P protein, and the degradation of p62/SQSTM1, but inhibited the cleavage of PARP and caspase 3 in a dose-dependent manner (Figure 7A). Interestingly, incubation with the pan-caspase inhibitor ZVAD-FMK in the presence of CQ reversed NDV-induced conversion of LC3-I to LC3-II and the degradation of p62/SQSTM1 in a dose-dependent manner. Next, we investigated if pretreatment with ZVAD-FMK reversed apoptosis after exposure to NDV in the presence of autophagy inhibitor or inducers (e.g., CQ, Rap and EBSS). Therefore, CEF cells were pretreated with CQ (50 μM), Rap (100 μM), or EBSS ( starvation) in the presence of ZVAD-FMK (40 μM) for 1h prior to NDV infection at an MOI of 1 and stained with annexin-V and PI at 24 and 48 hpi and analyzed by flow cytometry. As shown in Figures 4 and 7B, incubation with ZVAD-FMK decreased apoptosis in NDV-infected CEF cells without an autophagy inhibitor or inducer alone. Hence, our results indicated that the inhibition of apoptosis decreased apoptosis and enhanced host survival signals in response to NDV infection.

### Inhibition of autophagy enhances apoptosis *in vivo*

**Figure 8:**
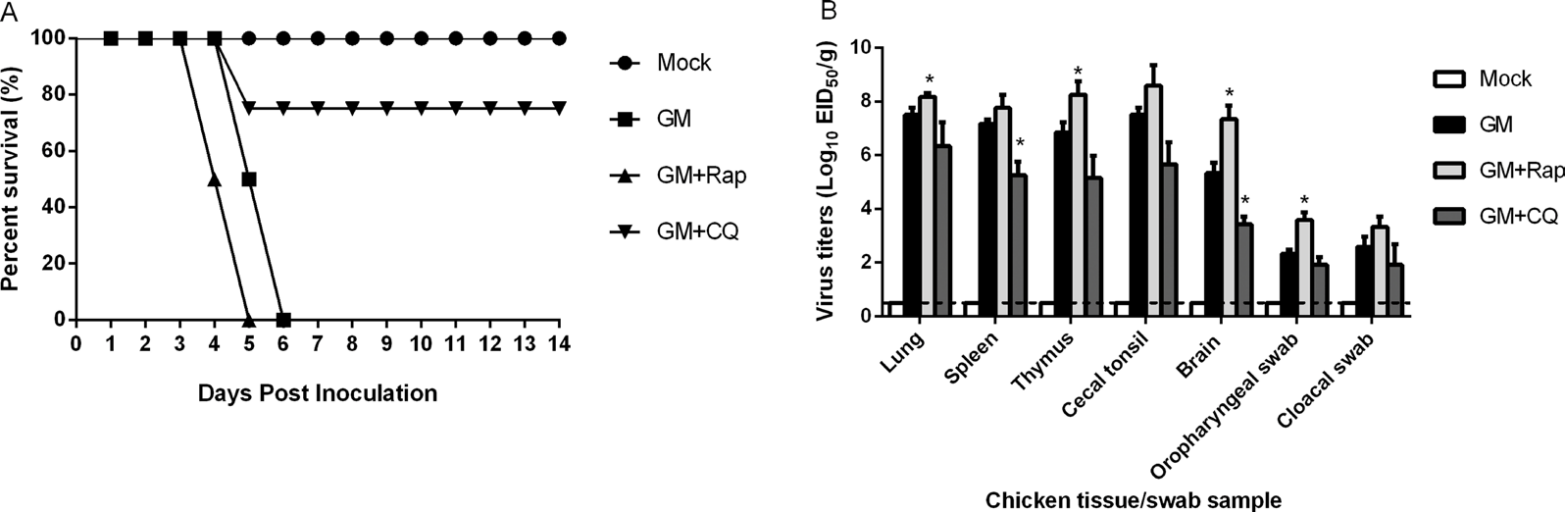
*In vivo* analysis of rapamycin (Rap), chloroquine (CQ) and mock-treated chicken groups infected with NDV Thirty-two 1d old SPF chicken were randomly divided into four groups and treated intracerebrally with CQ (40 mg/kg), Rap (2 mg/kg), or an equal volume of phosphate-buffered saline (PBS) 1 h before the administration of NDV and further at 12, 24, 36 and 48 h post-infection. Chicken were inoculated with 200 µL of 10^3^ 50% egg infectious dose (EID_50_) of the virus intranasally or intraocularly. Mock-infected chickens were injected intracerebrally with the same volume of PBS as a negative control. (**A**) The percent survival curve of chicken pretreated with CQ or Rap followed by NDV infection. (**B**) Virus titers in chicken tissues and swabs. At 3d post-infection, four chicken of each group were euthanized, and lung, spleen, brain, cecal tonsil, and thymus tissue, as well as oropharyngeal and cloacal swab samples were harvested and titrated for virus infectivity in SPF-embryonated chicken eggs.

The *in vivo* relationship between autophagy and apoptosis after NDV infection is not well known. Previous research reported that a goose-source NDV strain, Herts/33 (genotype II) induced autophagy *in vivo* and *in vitro*; CQ treatment inhibited virus replication in 7d old SPF chicken lung tissues but did not increase chicken survival rates; and treatment with Rap promoted virus replication in 7d old SPF chicken lung tissues without affecting survival rates [20]. Therefore, we investigated the *in vivo* role of autophagy in NDV replication and apoptosis in 1d old SPF chickens. We observed that pre-treating chickens with Rap accelerated the time of death during the 14d experimental period compared to NDV-infected chickens alone (Figure 8A, 8B). The viral titers of lung, brain, thymus, and oropharyngeal swab samples after exposure to Rap were higher than NDV-infected chickens alone at 3 dpi. In contrast, pretreating chickens with CQ increased survival rates (75%) during the 14d observation period. CQ treatment also reduced the viral titers of the spleen, lung, thymus, and brain tissues, as well as the oropharyngeal and cloacal swab samples compared to NDV-infected chickens alone at 3 dpi.

**Figure 9:**
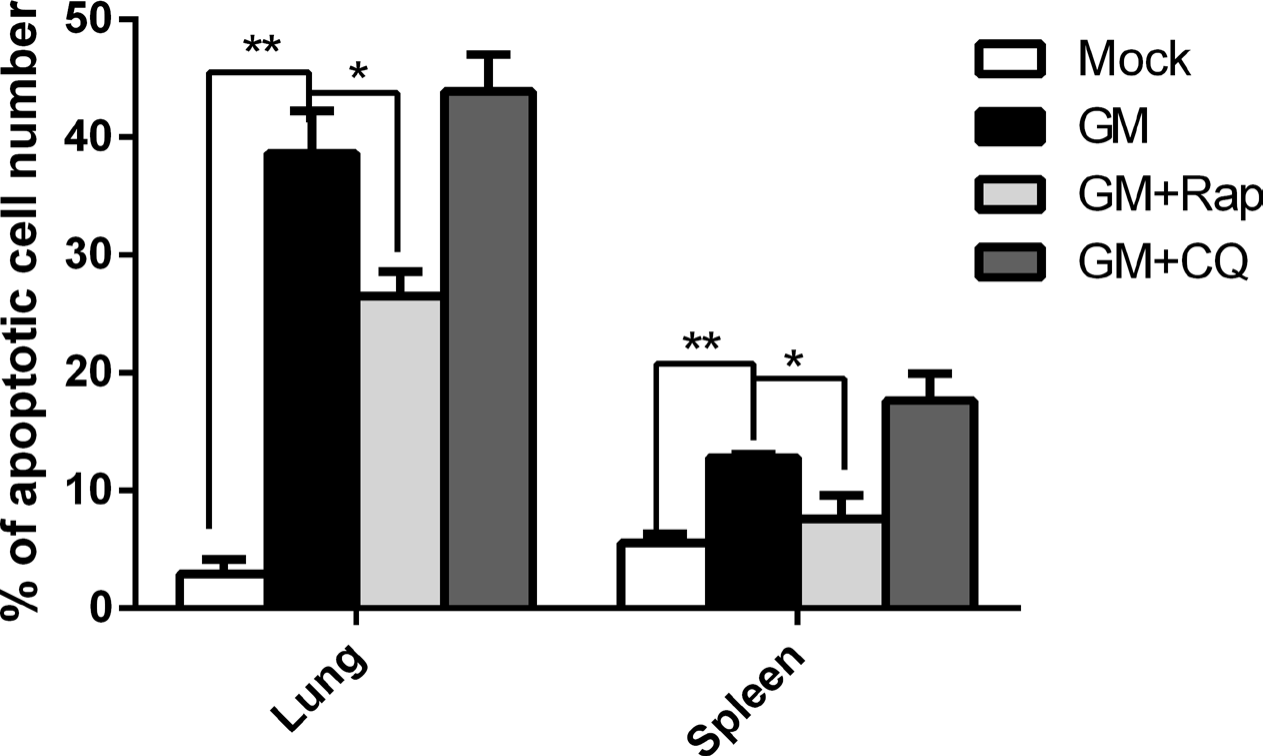
Regulation of autophagy affects apoptosis *in vivo* Chicken were pretreated with chloroquine or rapamycin as described in the materials and methods followed by NDV infection, after which lung and spleen tissues were harvested and ground for flow cytometry analysis using an AnnexinV–FITC/propidium iodide double-staining apoptosis detection kit. *Mean* ± *SD* of three independent experiments are shown. Similar results were observed in three independent experiments; *p < .05, **p < .01 (*t* test).

**Figure 10:**
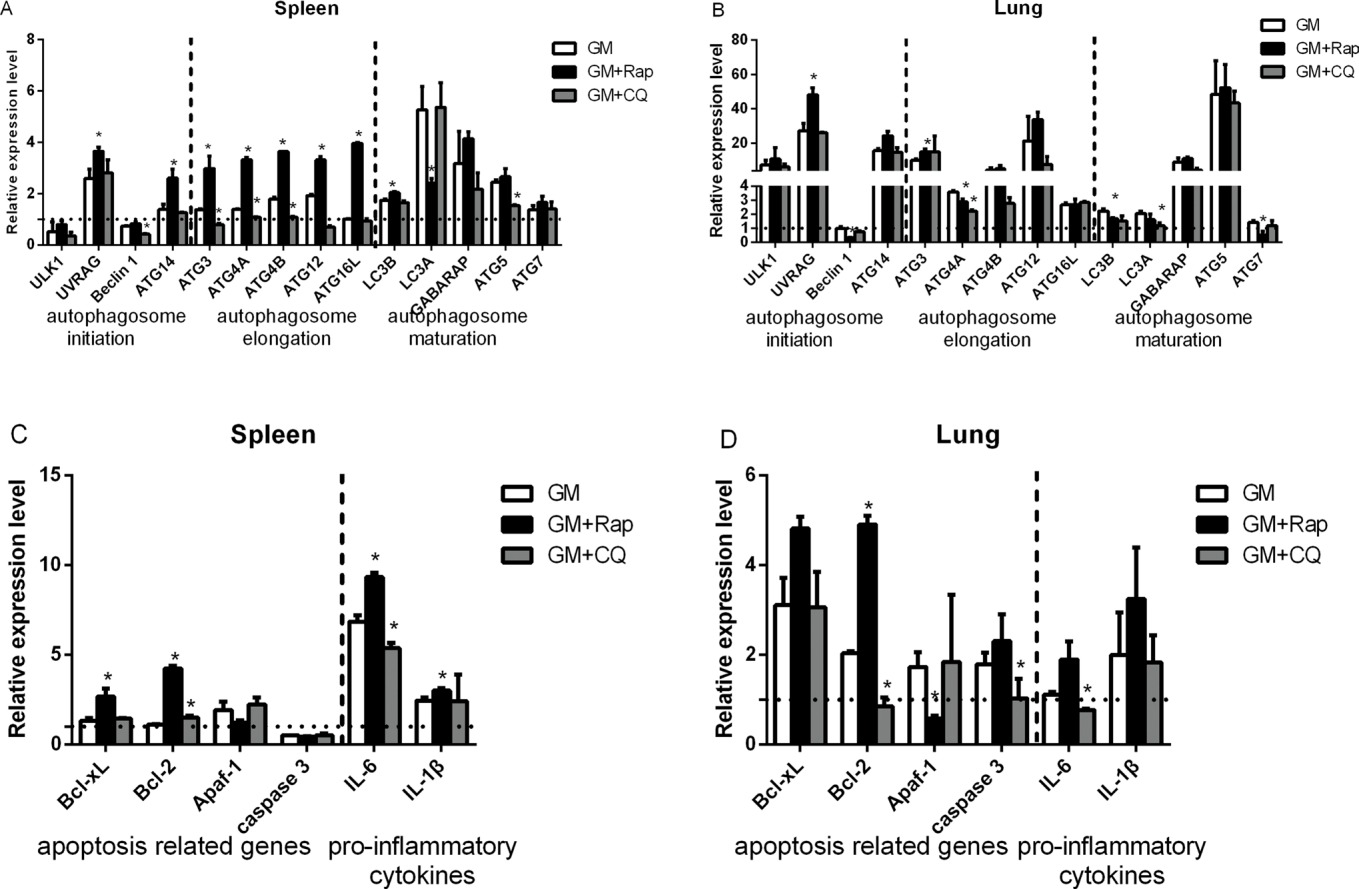
Relative expression of autophagy and apoptosis related genes, and proinflammatory cytokines in NDV-infected chicken spleens and lungs Chicken were pretreated with chloroquine or rapamycin as described in materials and methods followed by NDV infection, after which lung and spleen tissue was harvested and ground. Total RNA in the lung and spleen were extracted, reverse transcribed, and subjected to qRT-PCR to measure autophagy-related genes (**A** and **B**), apoptosis-related genes, and pro-inflammatory cytokines (**C** and **D**). *Mean* ± *SD* of three independent experiments are shown. Similar results were observed in three independent experiments; *p < .05 (*t* test).

To further investigate the role of autophagy in apoptosis *in vivo*, we harvested the lung and spleen tissues for flow cytometry analysis. As shown in Figure 9, treatment with Rap decreased the percentage of apoptotic cells, whereas treatment with CQ increased the percentage of apoptotic cells in the lung and spleen compared to infection with NDV alone. Further, we examined the levels of the autophagosome initiation complex (*ULK1*, *UVRAG*, *Beclin 1*, and *ATG14*), the autophagosome elongation complex (*ATG3*, *ATG4A*, *ATG4B*, *ATG12*, and *ATG16L*), and the autophagosome maturation complex (*LC3B*, *LC3A*, *GABARAP*, *ATG5*, and *ATG7*), as well as the apoptosis-related genes B-cell lymphoma extra large (Bcl-xL), B-cell lymphoma 2 (*Bcl-2*), apoptotic protease activating factor 1 (*Apaf-1*), and *caspase 3*, in addition to the proinflammatory cytokines interleukin 6 (*IL6*) and interleukin 1β (*IL-1β*) in the lung and spleen tissues following infection with NDV. We observed that *UVRAG*, *ATG14*, *ATG3*, *ATG4A*, *ATG4B*, *ATG12*, *ATG16L*, *LC3A*, *LC3B*, *GABARAP*, *ATG5*, and *ATG7*, of anti-apoptotic genes (i.e., *Bcl-xL* and *Bcl-2*), and of proinflammatory cytokines (i.e., *IL-6* and *IL-1β*) were upregulated in NDV-infected spleen and lung tissue compared to mock-infected chickens (Figure 10). However, *ULK1*, *Beclin 1*, and *caspase 3* were downregulated in the NDV-infected spleen, thereby suggesting that the spleen resisted the formation of autophagosomes. Furthermore, treatment with Rap increased *ATG*, *Bcl-xL*, *Bcl-2*, *caspase 3*, *IL-6*, and *IL-1β* in the spleen and lung. However, *LC3A* and *Apaf-1* in the spleen and *Beclin 1*, *LC3A*, *LC3B*, and *Apaf-1* in the lung were similar in Rap treated and NDV-infected only chickens. However, treatment with CQ downregulated *ATG, Bcl-xL*, *Bcl-2*, *IL-6*, and *IL-1β*, but not *Apaf-1* in the spleen and lung compared to NDV-infected only chickens. Therefore, our results demonstrated that autophagy was essential for *in vivo* NDV replication and apoptosis.

## DISCUSSION

Autophagy is a highly dynamic catabolic process involving degradation or recycling of cytoplasmic proteins and damaged organelles to maintain cellular homeostasis [25]. Autophagy is exploited by many viruses, including hepatitis C [30], influenza A [31, 32], dengue [33], classical swine fever [34], and NDV [20–22] to provide a physical scaffold for virus replication. Although previous studies have demonstrated that autophagy and apoptosis are both induced by NDV infection [19, 20], their interplay is not fully understood. In our study, we used the predominant genotype VII NDV strain (GM) in southern China to investigate the role of autophagy in NDV infection in chicken cells and tissues [3] and the relationship between autophagy and apoptosis.

The caspase family of proteins, including caspase 3 and PARP are involved in mediating the death-receptor and mitochondria–cytochrome pathways of apoptosis in host cells [6, 7, 9]. As reported previously for porcine reproductive and respiratory syndrome virus [35, 36], chikungunya virus [37], and enterovirus 71 [38], NDV triggers autophagy in chicken cells and inhibits caspase-dependent apoptosis as demonstrated by diminished cleavage of caspase 3 and PARP coinciding with autophagy induction by Rap and EBSS (Figure 6A and 6B). Also, the broad-specificity caspase inhibitor ZVAD-FMK inhibits cytokine activation and apoptosis initiation and execution in host cells [29]. It also reversed pro-apoptotic signals like conversion of LC3-I to LC3-II and degradation of p62/SQSTM1 and reduced apoptosis consistent with previous studies [37, 39]. Our results, therefore indicate that both autophagy and apoptosis are triggered by NDV infection in a caspase-dependent manner.

The induction of autophagy triggered by various viruses inhibits apoptosis and promotes virus propagation by prolonging the lifespan of host infected cells [40]. Our findings demonstrated that the inhibition of autophagy by CQ or the knockdown of Beclin 1 results in enhanced apoptosis as demonstrated by cleavage of caspase 3 and PARP, but also reduces viral titers in CEF cells (Figures 3 and 5). Likewise, *in vivo* treatment with CQ improves the survival rate of chicken by decreasing virus replication and shedding (Figure 8). These data indicate that the inhibition of autophagy promotes apoptosis as described for herpes simplex virus 1 and mouse herpesvirus 68 [41]. However, we could not demonstrate exclusive roles of autophagy and apoptosis in response to NDV infection.

The process of autophagy in mammalian cells involves six principal steps: initiation, nucleation, elongation, closure, maturation, and degradation [27]. We examined the mRNA levels of components of the autophagosome initiation complex (*ULK1*, *UVRAG*, *Beclin 1*, and *ATG14*), the autophagosome elongation complex (*ATG3*, *ATG4A*, *ATG4B*, *ATG12*, and *ATG16L*), and the autophagosome maturation complex (*LC3B*, *LC3A*, *GABARAP*, *ATG5*, and *ATG7*) in the lung and spleen following NDV infection. We observed that the expression levels of all *ATG* proteins except *ULK1* in the spleen and *Beclin 1* in the lung were upregulated when autophagy was induced by Rap (Figure 10). This suggested that autophagy was related to the chicken immune system or gender-specific differences, if not both [31, 42]. In contrast, the inhibition of autophagy decreased the expression levels of ATG proteins in the tissues targeted by NDV. Taken together, our results indicate that autophagy plays a crucial role in the response to NDV infection in host infected chickens.

In conclusion, we demonstrated that autophagy triggered by genotype VII NDV infection was essential for viral replication, NDV-induced apoptosis, and cell survival in chicken cells and tissues. These findings expand on the current understanding of the pathogenesis of NDV and provide new insights to control and prevent NDV infection.

## MATERIALS AND METHODS

### Ethics statement

Animal experiments were carried out in ABSL-3 facilities and were conducted in accordance with the guidelines of CDC’s Institutional Animal Care and Use Committee. The South China Agricultural University Experimental Animal Welfare Ethics Committee approved this study (permit no. 2015–03).

### Cells, virus, and experimental animals

Chick embryo fibroblasts (CEF) and DF-1 chicken fibroblasts (ATCC CRL-12203) were cultured and maintained in Dulbecco’s modified Eagle’s medium (DMEM, Life Technologies, Guangzhou, China) supplemented with 10% fetal bovine serum (FBS, Gibco, Carlsbad, CA, USA) with penicillin (100U/mL) and streptomycin (0.1mg/ml, Sigma-Aldrich, Shanghai, China) at 37°C in a humidified 5% CO_2_ incubator. The NDV strain Chicken/Guangdong/GM/2014 (GM), whose genotype (VII) is predominant worldwide [3], was isolated from dead chicken in China’s Guangdong Province and propagated in 9d old specific-pathogen-free (SPF) embryonated chicken eggs. Virus titers were determined by plaque assay as described previously [43, 44]. For *in vivo* studies, a group of 1d old SPF chicken were purchased from Guangdong Wens Dahuanong Biotechnology Co., Ltd (Yunfu, China) and housed in isolator cages

### Chemicals and antibodies

Rapamycin (Rap, R0395), chloroquine (CQ, PHR1258), rabbit polyclonal antibody of anti-p62/SQSTM1 (P0067), mouse monoclonal anti-GAPDH antibody (G8795), rabbit polyclonal anti-LC3B antibody (L7543), pan-caspase inhibitor ZVAD-FMK (V116) and annexin V–fluorescein isothiocyanate (FITC) double-staining apoptosis detection kit were purchased from Sigma–Aldrich. The rabbit polyclonal anti-Beclin 1 antibody (3738), the mouse polyclonal anti-poly(ADP-ribose) polymerase (PARP) antibody (9542), and the mouse polyclonal caspase-3 antibody (9662) were obtained from Cell Signaling Technology (MA, USA). Earle’s Balanced Salt Solution (EBSS,14155063) was purchased from Life Technologies. The mouse polyclonal anti NDV-P antibody was acquired from Key Laboratory of Animal Vaccine Development, Ministry of Agriculture (Guangzhou, China). IRDye 680 goat anti-rabbit IgG and IRDye 800CW goat anti-mouse IgG were purchased from LI-COR Biosciences (NE, USA). Beclin 1 small interfering RNA (siRNA) and a scrambled siRNA control were designed and synthesized by Life Technologies.

### Chemical treatment and virus infection of chicken cells

CEF and DF-1 cells were pretreated with dimethyl sulfoxide (DMSO), CQ, or Rap for 1h and then infected at a multiplicity of infection (MOI) of 1 with NDV, followed by adsorption in a 5% CO_2_ incubator at 37°C for 1 h. The optimal concentrations of CQ and Rap in our experiments were based on earlier research [20]. At indicated time points, cells and culture supernatants were harvested for further studies.

### Cell viability assay

Cell viability was measured by methylthiazolyldiphenyl-tetrazolium bromide (MTT) cell proliferation and cytotoxicity assay (Sigma Aldrich, USA) following the manufacturer’s protocol. Data represent average of three independent experiments.

### Transmission electron microscopy

CEF and DF-1 cells were infected with NDV at an MOI of 1 and harvested at 12 and 24h post-infection (hpi). The cells were fixed with 2.5% glutaraldehyde solution for 30 min at 4°C and post-fixed with 1% osmium tetroxide in 0.1 M sodium cacodylate buffer for 2 h at 4°C. Then, the cells were dehydrated with a graded series of ethanol and embedded in epoxy resin. Ultrathin (60 nm) sections were obtained, stained with uranyl acetate and lead citrate and observed and photographed under JEM-2010HR TEM. Mock-infected and EBSS-treated cells were used as negative and positive controls, respectively.

### Transfection of small interfering RNA

CEF or DF-1 cells were grown to 80% confluence in 60 mm cell culture dishes and transfected with Beclin1-specific or scrambled control siRNAs with Lipofectamine 3000 (Invitrogen, Carlsbad, CA, USA) according to the manufacturer’s instructions. Briefly, 1 µg of siRNA was diluted in 100 µL of reduced serum medium (Opti-MEM, Gibco). Then, 2.5 μL of Lipofectamine 3000 was directly pipetted into the diluted siRNA solutions, and gently shaken followed by incubation at room temperature for 20 min. The cell culture media in the 60-mm cell culture dishes were removed and washed thrice with DMEM and then replaced with 4ml of Opti-MEM containing the siRNA and the transfection reagents and further cultured at 37°C and 5% CO_2_. After 6 h, the cell culture supernatants were removed, and fresh DMEM containing 10% FBS was added to the cells for 24 h. Following infection with NDV for 1 h, infected cell lysates and supernatants were harvested for western blot analysis and plaque assay at 24 and 48 hpi, respectively.

### Western blot analysis

The cells were lysed in radioimmunoprecipitation assay buffer containing protease inhibitor tablets (Roche, 88266) on ice followed by ultrasonication and centrifugation at 14,000 × g for 10 min at 4°C. The protein concentration of cell lysates was quantified with the bicinchoninic acid protein assay kit (Pierce, 23227). Equal amounts of protein (25 µg) were denatured for 6 min at 95°C in 2× sodium dodecyl sulfate polyacrylamide gel electrophoresis (SDS–PAGE) loading buffer, separated by 15% or 12% SDS–PAGE gels, and electrophoretically transferred onto nitrocellulose membrane (Whatman Schleicher & Schuell, Dassel, Germany) using the semidry blotting method. Then, the blots were blocked for 2h at room temperature in Tris-buffered saline containing 0.1% Tween-20 and 5% bovine serum albumin and then incubated with primary antibodies at 4°C overnight, followed by incubation with the corresponding IRDye 680-conjugated anti-rabbit IgG or IRDye 800-conjugated anti-mouse IgG secondary antibodies (1:10,000) in the dark for 1h. After the membrane was washed thrice with tris-buffered saline containing 0.1% Tween-20, the blots were visualized with the Odyssey Infrared Imaging System (LI-COR Biosciences).

### Animal experiments

Thirty-two 1d old SPF chicken were randomly divided into four groups and treated intracerebrally with CQ (40 mg/kg), Rap (2 mg/kg), or an equal volume of PBS for 1 h before administration of NDV followed by further intracerebral inoculation at 12, 24, 36, and 48 hpi as previously described [20]. Pharmacologically treated chicken were inoculated with 200 µl of a 10^3^ 50% egg infectious dose (EID_50_) of the virus intranasally or intraocularly. Mock-infected chicken were injected intracerebrally with the same volume of PBS as negative control. All chicken were observed for morbidity and mortality in 12 h intervals for 14 dpi. At 3 dpi, four chicken of each group were euthanized and lung, spleen, brain, cecal tonsil and thymus tissues were harvested and titrated for virus infectivity in SPF-embryonated chicken eggs. Lung and spleen tissues, which are specific targets of NDV infection were ground and subjected to flow cytometry analysis of apoptosis. Also, RNA samples were subjected to qRT-PCR analysis to evaluate the mRNA expression levels of autophagy-related genes (ATG), apoptosis-related genes, and proinflammatory cytokines. Oropharyngeal and cloacal swabs samples were also collected from chickens at 3 dpi and suspended in 1ml PBS to detect NDV shedding.

### Flow cytometry analysis

Flow cytometry was performed to determine apoptosis using annexinV–FITC double-staining apoptosis detection kit according to the manufacturer’s protocol. First, CEF and DF-1 cells were pretreated with CQ, Rap or EBSS for 1h and then infected with NDV at an MOI of 1. Chicken were pretreated as described in the animal experiment. For flow cytometry analysis, cells were harvested and washed thrice with phosphate buffer saline (PBS), centrifuged, and suspended in 500 µL of 10× binding buffer, followed by treatment with 10 µl of FITC-labeled annexinV per sample for 5 min at room temperature. Then, the infected cells were stained with 5 µl of propidium iodide (PI) per sample for 10 min, followed by analysis in a FC500 flow cytometer (Beckman Coulter, Brea, CA, USA). AnnexinV-positive and PI-negative cell populations in the lower right quadrant of the AnnexinV versus PI FACS plot were regarded as apoptotic cells.

### RNA extraction and qRT-PCR

Total RNA of tissues were extracted with TRIzol reagent (Invitrogen, Carlsbad, CA, USA) following the manufacturer’s instructions. After treatment with DNase I (Takara), 1 µg of total RNA was reverse transcribed using the RevertAid First Strand cDNA synthesis kit (Takara, Dalian, China). Then, cDNA was used for real-time PCR amplification performed using the QuantiFast SYBR Green PCR kit (Qiagen) and a 7500 Fast Real-Time PCR system (Applied Biosystems, Rotkreuz, Switzerland). PCR primers used are summarized in Table 1. PCR protocol included one cycle of 95°C for 5 min followed by 40 cycles of 95°C for 15s and 60°C for 34s for amplification. Each tissue sample was tested in triplicate. Expression levels of target genes were normalized to a constitutively expressed housekeeping gene, GAPDH, and the fold change for each gene was calculated using the 2^*−ΔΔCT*^ method [45].

**Table 1:** Quantitative reverse transcription–polymerase chain reaction primers used in this study

Gene	Forward primer (5′→3′)	Reverse primer (5′→3′)	Product size (bp)	Accession number
GAPDH	CCTCTCTGGCAAAGTCCAAG	CATCTGCCCATTTGATGTTG	200	NM_204305
ULK1	CAGCCCATCCCCAGTGATTT	GCATACCGGAGACTCGAAGG	121	XM_015275648
UVRAG	TTTAACGCAGCCTGTAGCCA	GGGTATTGGTTAGGCTCCCG	57	NM_001030839
LC3A	GAATCCCACCCAGGCTTTCT	GTCTCCTGGGAAGCGTAGAC	80	XM_417327
LC3B	GTACGAGAGCGAGAAGGACG	AGACGGAAGATTGCACTCCG	75	NM_001031461
Beclin 1	TACGCAGGTCAGCTTTGTGT	ACATCATTCTGGCTGGTGGG	61	NM_001006332
ATG7	TCTGCCAAGTGTCTGTTGCT	CACCCCAACCCATCAGAGTC	57	NM_001030592
ATG5	ATACAGCCCTTCCTTGGAGC	TAACCCCATCCACAGTTGCT	66	NM_001006409
ATG14	GTGCTGACCTGGAAGACTCC	CCAGATCCGTCTCTTCGTCG	61	XM_426476
GABARAP	CTGCGAAGGGGAGAAGATCC	CGCTGTAGGCGATGTAGAGG	93	KP064313
ATG12	GCACCCGCACCATCCA	GAGGCCATCAGCTTCAGGAA	61	XM_003643073
ATG16L	TGCATCCAGCCAAACCTTTC	CGACGCTGGTGGCTTGTC	57	XM_003641751
ATG4A	CACAGCAGTGCACATTTGCA	CAGAGTCCTGCTGCGTTCCT	62	NM_001271986
ATG4B	CCCCGATGAAAGCTTCCA	GCTCAGCGATGCTCATTCTG	56	NM_213573
ATG3	GAACGTCATCAACACGGTGAA	TGAGGACGGGAGTGAGGTACTC	65	NM_001278070
Bcl-xL	CTCCATATCACCCCTGGCAC	CCCCCAGTTCACACCATCAT	127	GU230783
Bcl-2	GGATGGGATGCCTTTGTGGA	AGAGTGATGCAAGCTCCCAC	132	D11382
caspase 3	AAAGATGGACCACGCTCAGG	TCCGGTATCTCGGTGGAAGT	96	NM_204725
Apaf-1	GCCAAGCTACGGGCTCAAA	CGAACAACCAGACGGGAAAG	180	XM_015289686
IL-6	GAAGGAAGAGACTTCATTGCCTTGG	CTCTCCTCTCCAGTACGTCCTTCC	171	EU170468
IL-1β	CCACCTTTACCAGCTTCGAG	CCGTTCTTCATCCAGGTGAT	80	NM_204524

### Statistical analysis

All data are presented as *Mean* ± *SD*. Statistically significant differences were determined by a two-tailed independent Student’s *t* test using Prism 6.0 (Graph Pad Software Inc., San Diego, CA, USA) and *p* < .05 were considered statistically significant.
